# The complete mitochondrial genome of *Gyrodactylus kobayashii* (Platyhelminthes: Monogenea)

**DOI:** 10.1080/23802359.2016.1144102

**Published:** 2016-03-28

**Authors:** Dong Zhang, Hong Zou, Shun Zhou, Shan Gong Wu, Wen Xiang Li, Gui Tang Wang

**Affiliations:** a Institute of Hydrobiology, Chinese Academy of Sciences, Wuhan, PR China;; b University of Chinese Academy of Sciences, Beijing, PR China

**Keywords:** Gyrodactylus kobayashii, mitochondrial genome, Monogenea, phylogenetics

## Abstract

The complete mitochondrial genome of *Gyrodactylus kobayashii* was 14 786 bp in length, containing 12 protein-coding genes (lacking *Atp8*), 22 tRNA genes, two rRNA genes and two major non-coding regions (NC1 and NC2). The overall A + T content of mitochondrial genome was 71.6%. A close relationship between *G. kobayashii* and the three *Gyrodactylus* species (*G. salaris*, *G. thymalli* and *G. derjavinoides*) was uncovered in the phylogenetic tree based on amino acid sequences.

*Gyrodactylus kobayashii* was the most common *Gyrodactylus* species on the fins and gills of goldfish *Carassius auratus*. The worm was collected on goldfish from Wuhan (30°31'23”N, 114°23'01”E), China, and was identified by morphology and ITS molecular marker (Li et al. 2013).

The complete mitochondrial genome of *G. kobayashii* (GenBank accession no. KU057942) was sequenced by using long PCR and Sanger method of DNA sequencing. The circular mitogenome was 14 786 bp long and contained 12 protein-coding genes (PCGs, lacking *Atp8*), 22 tRNA genes, two rRNA genes and two major non-coding regions (NC1 and NC2) (Table 1). All the genes were transcribed from the same strand. The base composition was 41.9% T, 11.1% C, 29.7% A and 17.3% G. The disproportionally overall A + T content was 71.6%, which was higher than any of the three *Gyrodactylus* species (*G. salaris*, 62.3%; *G. thymalli* 62.8% and *G. derjavinoides*, 68.2%) (Huyse et al. 2007; Plaisance et al. 2007; Huyse et al. 2008). The gene order of *G. kobayashii* matched exactly with the three *Gyrodactylus* species.

The length of 12 PCGs was 9945 bp, with 71.6% A + T content. ATG was the unique start codon. *Nad5*, *Nad3*, *Nad2*, *Nad1* and *Atp6* appeared to use TAG as stop codon, whereas the rest of the PCGs used the stop codon TAA, and no premature stop codon (TA or T) was found. Total length of the 22 tRNA genes was 1436 bp, varying from 58 bp (*tRNA^Ser(AGN)^*) to 72 bp (*tRNA^Glu^* and *tRNA^Ala^*). All tRNAs could be fold into the conventional secondary structure, except for three unorthodox tRNAs, *tRNA^Ser(AGN)^*, *tRNA^Ser(UCN)^* and *tRNA^Cys^* lacked DHU arms. The *rrnL* and *rrnS* were 955 bp and 707 bp in size, respectively. They were flanked by *tRNA^Thr^* and *Cox2*, and separated by *tRNA^Cys^*, as demonstrated in the monopisthocotyleans (Huyse et al. 2007; Plaisance et al. 2007; Huyse et al. 2008; Perkins et al. 2010; Ye et al. 2014; Zhang et al. 2014a,b).

There were five cases of overlapping regions within the mitogenome. The overlap between *Nad4*L and *Nad4* was common in metazoan mtDNAs (von Nickisch-Rosenegk et al. 2001), with the exception of *Benedenia hoshinai* and *B. seriolae* (Perkins et al. 2010). There were 21 short intergenic regions ranging from 1 bp to 111 bp. In addition, the two long non-coding regions, NC1 (between *tRNA^Phe^* and *Atp6*) and NC2 (between *tRNA^Met^* and *tRNA^Ser(UCN)^*) were 778 bp and 783 bp long, 67.0% and 68.2% for the AT content, respectively. The high similarity over 686 bp sequences was found between NC1 and NC2, with the differences in eight substitutions and three indels.

The phylogenetic analysis was performed with 2858 homologous concatenated amino acid sequences representing 12 protein-coding genes from nine available mitochondrial genomes and *G. kobayashii* mitogenome (this study), implementing maximum-likelihood (ML) and Bayesian inference (BI) analyses. Both the phylogenetic methods produced the same tree topology in the branching patterns. *Gyrodactylus kobayashii* was closely relate to the three *Gyrodactylus* species with extremely high bootstrap resampling (ML) and posterior probability (BI) values (Figure 1).

**Figure 1.**
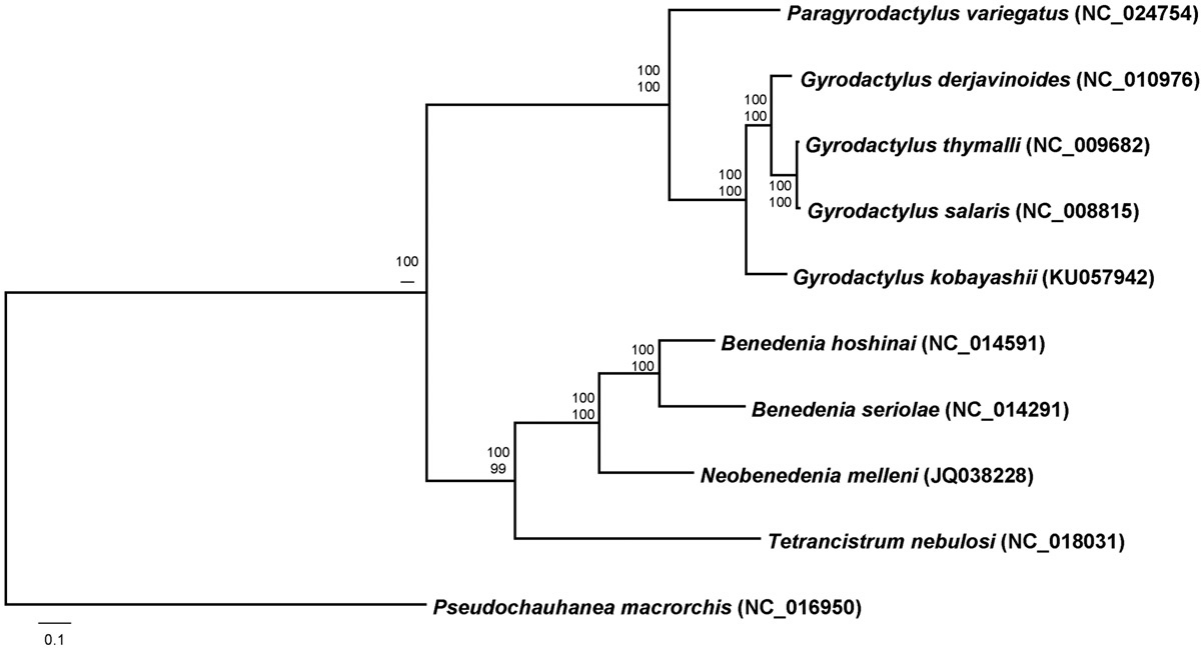
Phylogenetic tree of *Gyrodactylus kobayashii* and selected monopisthocotyleans based on the concatenated amino acids representing 12 mitochondrial protein-coding genes. The MtZoa model for maximum-likelihood analysis and MtREV model for Bayes analysis are selected according to AIC criterion. Scale bar represents the estimated number of substitutions per site. The numbers at the nodes indicate posterior probability (upper value) and bootstrap probability (lower value).

**Table 1.** Organization of the mitochondrial genome of *Gyrodactylus kobayashii*.

Gene/region	Position		Size	Intergenic nucleotides	Codon		Anti-codon
	Start	Stop			Start	Stop	
*Cox3*	1	639	639		ATG	TAA	
*tRNA^His^*	646	712	67	6			GTG
*Cytb*	716	1789	1074	3	ATG	TAA	
*Nad4L*	1789	2037	249	–1	ATG	TAA	
*Nad4*	2010	3218	1209	–28	ATG	TAA	
*tRNA^Phe^*	3221	3285	65	2			GAA
NC1	3286	4063	778				
*Atp6*	4064	4576	513		ATG	TAG	
*Nad2*	4585	5442	858	8	ATG	TAG	
*tRNA^Val^*	5443	5506	64				TAC
*tRNA^Ala^*	5508	5579	72	1			TGC
*tRNA^Asp^*	5582	5646	65	2			GTC
*Nad1*	5647	6534	888		ATG	TAG	
*tRNA^Asn^*	6534	6600	67	–1			GTT
*tRNA^Pro^*	6601	6663	63				TGG
*tRNA^Ile^*	6660	6724	65	–4			GAT
*tRNA^Lys^*	6726	6789	64	1			CTT
*Nad3*	6791	7138	348	1	ATG	TAG	
*tRNA^Ser(AGN)^*(S1)	7139	7196	58				GCT
*tRNA^Trp^*	7206	7270	65	9			TCA
*Cox1*	7275	8822	1548	4	ATG	TAA	
*tRNA^Thr^*	8831	8895	65	8			TGT
*16S rRNA*	8895	9849	955	–1			
*tRNA^Cys^*	9854	9914	61	4			GCA
*12S rRNA*	9915	10 621	707				
*Cox2*	10 622	11 203	582		ATG	TAA	
*tRNA^Glu^*	11 315	11 386	72	111			TTC
*Nad6*	11 390	11 872	483	3	ATG	TAA	
*tRNA^Tyr^*	11 893	11 960	68	20			GTA
*tRNA^Leu(CUN)^*(L1)	11 967	12 032	66	6			TAG
*tRNA^Gln^*	12 041	12 104	64	8			TTG
*tRNA^Met^*	12 105	12 169	65				CAT
NC2	12 170	12 952	783				
*tRNA^Ser(UCN)^*(S2)	12 953	13 011	59				TGA
*tRNA*^Leu^*^(UUR)^*(L2)	13 014	13 081	68	2			TAA
*tRNA^Arg^*	13 085	13 151	67	3			TCG
*Nad5*	13 152	14 705	1554		ATG	TAG	
*tRNA^Gly^*	14 718	14 783	66	12			TCC
	14 787	14 786		3			
